# Analyzing and mitigating the risks of patient harm during operating room to intensive care unit patient handoffs

**DOI:** 10.1093/intqhc/mzae114

**Published:** 2024-12-19

**Authors:** Nara Regina Spall Martins, Edson Zangiacomi Martinez, Cláudia Marquez Simões, Paul Randall Barach, Maria José Carvalho Carmona

**Affiliations:** Faculdade de Medicina, Universidade de São Paulo (USP), Av. Dr. Arnaldo, 455 - Sala 4107, São Paulo, São Paulo 01246-903, Brazil; Faculdade de Medicina de Ribeirão Preto, Universidade de São Paulo (USP), Avenida Bandeirantes, 3900 Bairro Monte Alegre, Ribeirão Preto, São Paulo 14049-900, Brazil; Faculdade de Medicina, Universidade de São Paulo (USP), Av. Dr. Arnaldo, 455 - Sala 4107, São Paulo, São Paulo 01246-903, Brazil; School of Medicine, Thomas Jefferson University, 901 Walnut St Ste 10, 19107, Philadelphia, Pensilvânia 19107, United States of America; Faculty of Medicine, Sigmund Freud Private University, Freudplatz 3, Vienna A – 1020, Austria; Faculdade de Medicina, Universidade de São Paulo (USP), Av. Dr. Arnaldo, 455 - Sala 4107, São Paulo, São Paulo 01246-903, Brazil

**Keywords:** risk management, process mapping, hand-off, qualitative research, failure modes and effects analysis (FMEA)

## Abstract

Patients continue to suffer from preventable harm and uneven quality outcomes. Reliable clinical outcomes depend on the quality of robust administrative systems and reliable support processes. Critically ill patient handoffs from the operating room (OR) to the intensive care unit (ICU) are known to be high-risk events. We describe a novel perspective on how risk factors associated with the process of patient handoff communication between the OR and the ICU can lead to flawed communication, degraded team awareness, medical errors, and increased patient harm. Data were collected from two semi-structured focus groups using a five-step risk management approach at a tertiary hospital in São Paulo, Brazil. We conducted a failure modes and effects analysis (FMEA) with multidisciplinary healthcare providers consisting of attending physicians, anesthesiologists, nurses, and physiotherapists involved in patient handoffs. We analyzed the results using a similitude analysis to evaluate the effectiveness of implementing this novel risk management approach. We identified the handoffs risks associated with patients, staff, institution, and potential financial risks. The FMEA identified 12 process failures and 36 causes that generated 12 consequences and pointed to robust needed preventive measures to mitigate handoff risks. The clinical teams reported that this approach allowed them to see the process more completely as a whole not only in their narrow silos, thus understanding the enablers and difficulties of the other team members and how this understanding can shed light on their mental models, actions, and the process reliability. Teams identified key steps in the OR to ICU handoff process that are prone to the highest hazards to patients, the hospital, and staff, and are currently targeted for process improvement. Evidence-driven recommendations intended for reducing the risks associated with patient handoffs are presented. Implementing a dynamic risk management, interdisciplinary approach was used to redesign the OR to ICU patient handoff approach around the patient’s and clinician’s needs. The risk management program helped healthcare providers identify handoff steps, highlighting risky handoff process failures, making it possible to identify actionable failures, consequences, and define preventative action plans for mitigating the risks to improve the quality and safety of patient handoffs.

## Introduction

The World Health Organization (WHO) estimates that more than 42 million patients suffer from adverse events annually [1]. Adverse patient events result in tragedies for patients and their families, damage the public trust in healthcare and organizations, add costs and lead to destructive malpractice litigation. In the UK, a patient harm incident is reported to occur every 35 s on average, and up to 15% of hospital spending in Europe goes toward addressing safety accidents. A study of 11 hospitals in Massachusetts identified adverse events in almost one in four admissions, and, approximately a quarter of the events were preventable [1, 2]. Many of these adverse events were attributed to failures in the handoffs of patient care [1]. Remarkably, 45% of European Union-based hospital staff think that handoffs and transitions are adequate [3].

The Critical Safety Study found that adverse events in intensive care unit (ICU) occur at a rate of 81 per 1000 patient-days, and serious errors occur at a rate of 150 events per 1000 patient-days, supporting previous studies that most ICU patients experience preventable harmful events [4]. The study found that the incidence of adverse events was 20.2% (with 13 deaths, and 55% of events preventable).

Effective OR to ICU patient handoffs must include not only OR details but also anticipatory guidance that allows ICU teams to prepare and continue providing immediate postoperative care and to anticipate, detect, and facilitate timely management of postoperative complications [5]. However, the OR to ICU remains fraught with challenges, as it requires coordination of the physical and organizational transfer of the patient and the knowledge and responsibility transfer between multiple clinicians, performed in a busy, understaffed clinical environment.

While all organizations assess risks, many do not apply an explicit, standardized risk assessment and management system to help control, prevent, and monitor dynamic risks. A safety management system allows for the threat and error mitigation of these events, improved incident management and provides a secure foundation for risk mitigation decision-making and planning [6].

The aim of the study was to gain insights into, and evaluate how, implementing a novel risk management approach targeted at the handoff process between the OR and the ICU teams in a large tertiary hospital, can identify potential failures and generate preventative, improvement solutions for harm prevention.

## Methods

### Study design and setting

**Figure 1:**
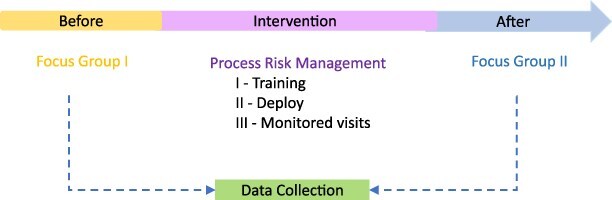
Study design and research methodology

We conducted a prospective, qualitative intervention on patient handovers between the OR and the ICU staff of a tertiary academic medical center. The study design applied action-research methodology for improving clinical practice [7] (Fig. 1).

### Multidisciplinary team

The ISO 31000:2018 standard recommends that risk analysis be carried out by a multidisciplinary team containing at least one employee from each process activity with skill, experience, and competence, allowing stakeholders to be properly represented [8].

Of the 185 multidisciplinary healthcare providers (HCWs) involved in conducting patient handovers, 13 HCWs were selected for deployment of the process risk management by the managers of the areas involved. The team was composed of at least two representatives from each clinical area as shown in the following table (Table 1).

**Table 1.** Study multidisciplinary team

Area	Position	*N*
OR	Nurse	2
	Anesthesiologist	2
ICU	Adult intensive care physician	2
	Pediatric intensive care physician	2
	ICU nurse	3
	Physiotherapist	2
Total providers		13

The teams participated in three activities:

(a) Meetings—The team was divided into two groups that met twice a week for 8 weeks. Each meeting lasted 2 h, totaling 32 h.

(b) Trainings—All team participants received theoretical and on-site training that included lectures during the training meetings about the concepts of process risk management, regulatory requirements, and the tools used for process analysis. The training was carried out during the weekly meetings to give the participants the necessary knowledge to implement the methodology and apply concepts to other areas of the hospital. In total, the training required 8 h.

(c) Ethnographic observations—A researcher accompanied by a team member, made regular visits to the OR and ICU areas and observed dozens of patient handoffs and talked to many staff to clarify doubts that arose during the meetings.

### Deployment of process risk management

**Figure 2:**
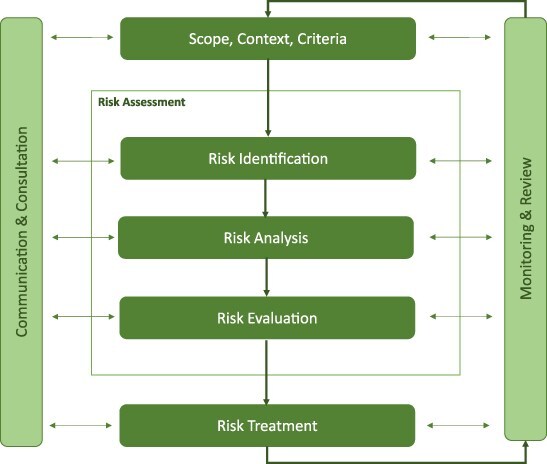
Risk management process—adapted from International Organization for Standardization ***ISO 31000:2018*** [8]

The risk management approach followed the guidelines determined by the international ISO 31000:2018 standard and is presented in Fig. 2. The steps include:

(a) Communication

We discussed and collected HCW perceptions regarding patient’s handover risks considered during all phases of the study [8].

(b) Establish the context

We analyzed staffing priorities, regulatory norms, legislation, technology, key factors, and organizational structure that impact the care processes were discussed and evaluated [8].

(c) Risk assessment process

We used a series of interlocking tools, and focused on care objectives, organization’s culture, computer support, and technical knowledge [1, 8, 9]. The steps included:

- Risk identification: We identified the sources of risks, impact areas, events, their causes, and potential consequences for each process [8, 10]:
  - Process mapping: A representation of the process through the eyes of HCW using a flowchart was done containing all steps, including the definitions of the beginning and end of processes, the products of the process, and points of intersection with other organizational processes [11]. The process maps allowed the teams to identify redundancies, inefficiencies, misunderstandings, delays, and decision points. We brainstormed and encouraged the HCW involved to provide a greater number of ideas in a short period of time [1, 9, 11, 12].
  - Risk analysis and evaluation: We quantified and measured the risks to assist the clinical decision-making. Many techniques can be used for these steps, as suggested in the ISO 31010:2019 standard . In this study, we used the failure modes and effects analysis (FMEA) method [8, 10] where the severity, criticality, frequency, and detectability of each risk were analyzed. Based on these elements, we obtained the RPN (Risk Priority Number). The higher the RPN, the greater the chance of the failure mode occurring, and the more justifiable the adoption of countermeasures to control these risks. The HCW team evaluated detailed process maps, current and historical quality indicators of the institution, and the areas that are known to affect patient handovers. Participants were invited to reflect on the possible effects of a failure, whether they were linked to patients, the hospital, or staff [1, 9, 10, 13–15].

(d) Risk treatment

The team defined the actions to mitigate the patient risks and considered the following [1, 8–10]:

- Discontinuing or not starting activities that may generate risks;
- Removing the source of the risks;
- Altering the probability or consequences;
- Sharing the risks with other parties; and
- Retaining the risks by conscious and well-founded decisions.

(e) Monitoring and critical analysis

The key responsibilities and frequencies of risk reassessment were defined

[1, 8–10].

### Evaluation of process risk management

#### Data collection

Two focus groups were conducted using a semi-structured protocol in Portuguese that was piloted, for approximately 60 min, with 100% of the HCW who participated in the intervention and varied in size from 2 to 6 participants per group. The focus groups were grounded in the systems engineering SEIPS framework [16], led by an experienced moderator, who put people at ease by encouraging participation and kept the discussion flowing and on message [17].

The topics that guided the focus group protocol were the following:

1. Knowledge and experience with process risk management concepts;
2. Experiences with recent patient handovers;
3. Perceptions about handovers in general (beliefs, norms, assumptions, methods, tools, barriers, and facilitators);
4. Perceptions about role taking, tasks, and responsibilities; and
5. Suggestions for improving patient handovers.

The focus groups were audio-recorded in addition to the moderator taking notes about the non-verbal language [18]. At the end of each focus group, the moderator summarized the information and allowed participants to reflect and comment on the accuracy and validity of the summary. The data were collected between February 2020 and April 2020.

The first focus group was conducted before the intervention to assess prior knowledge of the team and how they evaluated risk management about the processes in their clinical work. Participants were also asked to complete a questionnaire containing socio-demographic information [19]. The second focus group was conducted after the program implementation, to evaluate whether the method surfaced new potential failures for preventive action.

#### Data analysis

The participants’ characteristics were analyzed using descriptive statistics. All interviews were transcribed verbatim according to a standardized format [19, 20]. The transcription corpus was analyzed and each response to a question constituted a text. The entire corpus was coded and analyzed using IRaMuTeQ qualitative analysis software [19, 21]. The results were proofread for typos, spelling, grammatical errors, and standardization of acronyms to select relevant words and assigned weights according to their importance.

The focus group data were analyzed using a similitude analysis [20, 22–24]. Similarity is calculated according to the vectorized terms (selection of relevant words and weighting them according to their importance). Two vectorized terms that share the same vocabulary (i.e. similar word competition patterns) are interpreted as being semantically close to each other.

This analysis makes it possible to identify co-occurrences between words, providing indications of the connection between them and helps identify the structure of the content of a textual corpus. It is also possible to identify common parts and specificities according to the descriptive variables identified in the analysis.

The results are presented graphically, where the size of blue circles is proportional to the scores of the evoked words. The edges of the graphic indicate the values of the association between words. The analyses point to a set of words that are strongly associated with parts of speech clustered together.

## Results

### Deployment of process risk management

The intervention generated documents containing information and analysis of the process and its risks. These results are presented below following the steps recommended by the ISO 31000:2018 standard.

Step 1. Communication

Communications took place via standard emails and WhatsApp messages, especially for sending training materials, analyzed documents, and discussions between the multidisciplinary team, for facilitating the exchange of relevant, accurate, and understandable information (Supplementary Appendix 1).

Step 2. Establishing the context

The context analysis was carried out with hospital managers before the meetings to evaluate the internal norms, key factors, organizational structure, and information systems. The external context analysis was carried out through an analysis of the Brazilian regulatory standards and legislation, including a review of national accreditations, legislation, and guidance from regulatory bodies.

Step 3. Risk assessment

The team validated the overall process at the end of each stage and suggested process improvements using the following steps:

- Risk identification

Process Mapping: The team used the process representation with the help of colored postcards that allowed the participants to build, validate, draw, and redraw the process maps with greater agility and assurance (Supplementary Appendix 2).

Identification of Failure Modes: We used brainstorming to stimulate the review of the process maps, and participants were invited to raise as many potential failures and their effects on patient care as freely, quickly, and without judgement (Supplementary Appendix 3).

- Risk analysis and evaluation

We developed the FMEA which identified 12 potential failures related to 36 causes, and that generated 12 distinct consequences. For each combination of factors, we determined the preventive measures to control these patient risks. Each participant scored the risks to analyze the severity, frequency, and detectability of the risks. The team considered current and historical institutional quality measures.

Some potential failures identified by the team included a lack of or incomplete patient information at the various points of contact between teams or during shift changes, failure or lack of preanaesthetic assessment, and failure to admit the patient.

The analysis of causes included machines, methods, materials, measures, environment, and procedures. Some examples of causes are alignment of conduct, culture, incorrect definition of type of surgery, delays in releasing beds, filling in forms, system unavailability, limited resources, etc.

Among the various consequences raised related to a delayed surgery with patient harm associated were as follows:

- Suspension of surgery with harm to the patient;

- Increased bed preparation time;

- Lack of operating room (OR) rotation;

- Financial consequences;

- Contagion from other patients;

- Adverse events to the patient;

- Employee—biological risk (example: isolation, accidents);

- Legal proceedings; and

- Negative impact on the institution’s image.

Step 4. Risk treatment

When the risk was defined as high, the team proposed a plan with 23 preventive actions to improve the quality of the process.

Of the action plans suggested by the team, some involved the redesign of the handoff processes and investing heavily, so there are plans that can be implemented quickly and others that need to be implemented over the long term. In the latter case, in order to implement them, there is a need for approval from the appropriate managers.

Examples of action plans include implementing known handoff methods such as I-PASS or ISBAR, training staff and residents, acquiring mobile equipment, improving bed release logistics, among others.

Step 5. Monitoring

The team set deadlines for reassessing the risks, effectiveness of the proposed controls, and monitoring of changes to the plans.

### Evaluation of process risk management

#### Demographics

The study participants were composed of 85% women, 69% of the employees had university degrees, 72% worked exclusively at the hospital, and 77% were frontline providers not in management positions.

#### Focus groups

The final focus group corpus consisted of 429 texts, 2056 different words, and 1047 words. The participants reported that the process maps allowed them to see and appreciate the process as a whole more fully and not only the parts of the process they worked in. This allowed them to visualize the enablers and difficulties of the other clinical areas and how it affected the work of the next clinical areas and the overall work process.

**Figure 3:**
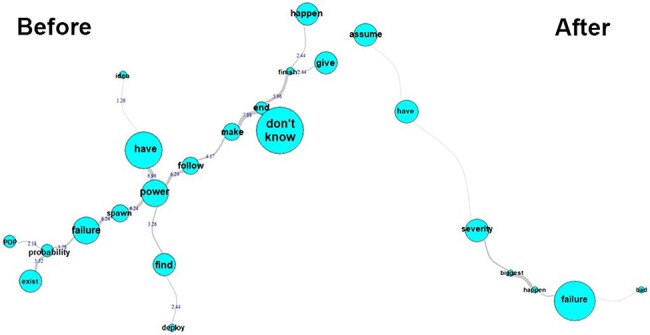
Similitude analysis (Q10) representing the process risks before and after

We found that most HCW had no experience in reflecting on, and constructing a process map, raising potential failures, identifying risks, and suggesting action plans for service improvement. Figure 3 shows what we found when we evaluated the team’s knowledge about process risks. Before applying the methodology, the most relevant and frequent term was “I don’t know,” which suggests that most participants were not trained and did not know what organizational and patient process risks were.

The participants said they understood the need to “assume” the risk, assess the levels of the risk “severity,” and the key was to follow up on the identified “failure” risks.

The participant teams reported the possible consequences (effects) before implementing the risk methodology, where the central term was the “patient,” which is also a term of great relevance in the comments of the participants. It is connected to the terms “failure,” “damage,” and “worst.”

**Figure 4:**
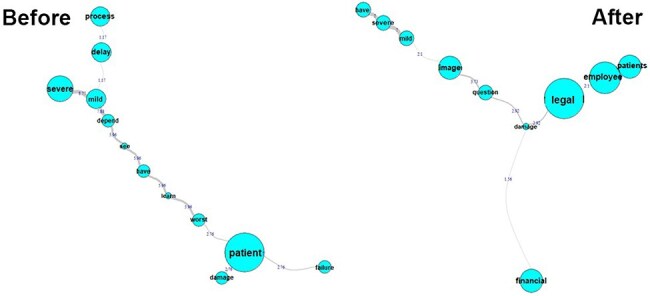
Similitude analysis (Q12)—representing the consequences before and after

After applying the risk analysis, the HCW quickly realized that in a process, there are other risks besides those related to the patients that needed to be evaluated and managed, such as risks to the employees, institution’s image, and financial risks. They emphasized that there are many potential “mild” and “severe” consequences (Fig. 4).

When the participants were asked about the benefits and difficulties of using this tool, two themes emerged and they are visualized in the figure below.

- The term “know,” which is connected to “seeing/achieving” (the “instrument,” the “final product”), to the term “tool” (which is connected to “apply” to the “process use” in “failures”), and to “vision” (“clear”).
- The term “put,” which is connected to the terms “happen,” “action plan” (in which the participants talk about the “need” to “punctuate”) and the term “form” (which during the speech talked about “problem identification,” interesting the “frontline participation” although it seems “complex” to join a multidisciplinary team in this kind of activity is essential).

The term “relevance” is a “process” that was mentioned several times in reference to the possibility of seeing and analyzing the risks.

**Figure 5:**
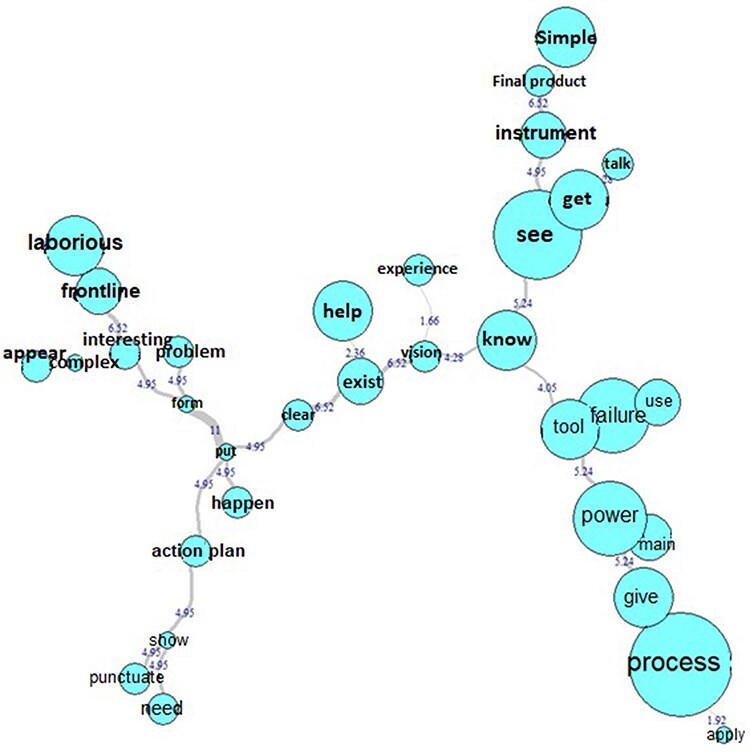
Similitude analysis (Q21)—the benefits and difficulties of process risk management

A point that captured our attention was the ambiguity of the participants who, at the same time, commented on how “laborious” the tool seemed to those who were using it for the first time, but that it was also “simple” to apply and incredibly valuable (Fig. 5).

The participants unanimously recommended the methods be applied to other hospital processes. They stressed the importance of “front-line participation,” and bringing together a multidisciplinary team for this type of activity and considered that the support of management was fundamental for effective implementation (Table 2).

**Table 2.** Themes, categories, and representative quotes related to patient handovers

Categories	Moment	Representative Quotes
Process risks	Before	‘I really never think I never heard about it’ (ID25).
	After	‘If the risk of that process we assume it or not, we decide by having the study’ (ID 15).
Consequences (effects)	After	‘It can be from image it can be from damage to the team a damage to the patient a damage to the institution. that we end up thinking only in the biological only in the patient in the team, financial risk sometimes a life issue sometimes comorbidity’ (ID 25).
Benefits of implementing process risk management	After	‘If we start applying this to everything, we will solve the world’ (ID17).
		‘If we could really discuss at least two processes a year, two processes every three months, and review something, I think it would refine our work a lot and improve our experience inside the hospital’ (ID11).
		‘What are the main points that we need communication that is where we had a greater chance of failure. He showed where the main failures are most important and essential to improving communication’ (ID21).
		‘Big benefit was seeing the other’s view better and getting to know one’s own’ (ID25).
Would recommend the methodology applied for risk management in other processes	After	‘It needed to be more widespread in other areas, it needed to be more widespread in the hospital’ (ID11).
		‘I think that we need to train those of us who are on the assistance front line’ (ID12).
		‘One thing that I thought was cool because then we started to understand what in the other area’ (ID15).
		‘For me, it was a learning experience … it brought a different view of how to analyze’ (ID21).
		‘Much easier to visualize where we can act or not’ (ID23).
		“To see if it is viable, because if the manager does not support us … it will depend on the managers “(ID24).
		‘We saw the consequences of something that is usually small, and that can have much greater consequences than I imagined’(ID25).
		‘I think it is important to always work with the involved people’ (ID26).

## Discussion

### Statement of principal findings

Implementing innovative risk management protocols allowed hospital teams to evaluate more effectively the risks of patient handoffs in a comprehensive and reflective manner. While this topic has been researched for many years, patient handoffs continue to pose major risk challenge to hospitals. This is the first time the ISO 31000:2018 guideline approach is used to assess the risks in patient handoffs [8]. Our work provides a novel perspective and approach on how to better identify potential failures at each step of the handoff, the causes, consequences, and existing controls. The occurrence of failure modes were evaluated and action plans were defined for the risks that justify the adoption of risk control measures.

### Interpretation within the context of the wider literature

Patient handoffs from the OR to the ICU continue to pose significant risks to patients and are highly variable. Ensuring the safety, effectiveness, and continuity of care during patient handoffs remains a main concern for hospital care providers [9]. We found that the challenges in implementing robust OR to ICU patient handoffs requires understanding the interdisciplinary workflow processes, organizational priorities, outputs, and staffing risks critical for hospitals to deliver postoperative, safe, and high-quality care. Thus, understanding and assessing the patient risks becomes essential for healthcare facilities to function effectively and safely.

Implementing a novel and reliable risk management approach involves a series of well-defined steps that can assist multidisciplinary teams to carefully examine each step of the workflow process by assessing the potential failure modes, causes, consequences, and existing controls [1, 8–10, 13, 25]. Our study invited the professionals to step outside their comfort zones and think about the current process as a whole, not restricting themselves to their activities following the proposed method. The proposed method was to take the necessary actions to mitigate the risks identified in a preventive manner.

Many studies address patient transfers from different perspectives (such as at the change of shift duty, at transfer between areas, at the transfer of units, and during patient discharge), yet these studies are commonly evaluated in a one-off manner at the time of transfer only [26, 27]. Our research has shown that failure at the time of patient transfer may occur due to failures at several “points” of the previous process, and that these vulnerability points can influence the other points downstream contributing to cascading failures at the time of patient transfer. This implies that safe patient transfer depends on the level of experience, knowledge of the health professionals involved, and the use of standardized tools, but also on the quality and transparency of the information received from the previous upstream steps in the clinical workflow.

Organizations could benefit from developing training programs in risk assessment and management, enabling HCW to identify critical gaps and help them improve their daily workflows [9]. The issue of training is not restricted to risk management, but also needs to be constantly updated for safe patient handoffs. Many physician residents stated that hands-on experience was the most effective way to understand and mitigate patient risks [26]. The development of theoretical training and the practical implementation of the risk analysis methodology in our research allowed the participants to visually assess patient risks, interact and understand in detail the steps before and after their activities, and propose process improvements using new ways to enhance the reliability of OR to ICU handoff process.

We identified few employees that had participated in risk mitigation initiatives, and these were mostly from managerial positions. The participants understood that the involvement of a larger number of frontline HCW professionals was critical for the risk analysis success. This understanding is reinforced by Berwick who states that “Without renewed board and executive leadership and accountability for safety and without concerted, persistent investment in and monitoring of change, a summary study 34 years from now may again look all too familiar, with millions upon millions of patients, families, and health care staff paying the price for inaction” [28].

### Implications for policy, practice, and research

The participants commented that the results “opened their eyes” and helped them move away from their traditional thinking about risk and toward appreciating the entire patient flow process as a whole, not restricting themselves to their siloed work activities.

Hospital managers coordinate hospital processes to ensure patient safety. Ensuring that employees directly involved in the process join in implementing risk management steps is fundamental as they know the process in detail and that can impacts the likelihood that proposed changes are well received and sustained.

### Strengths and limitations

A key strength of our study is the implementation of a practical and reproducible structured risk analysis methodology that makes it possible to assess and take actions on all risk types (for patients, employees, financial, legal, brand, and others) carried out by and with the active support of the “front line” employees.

This research proved to be important for a complete understanding of the stages of the process in the patient handoffs, the interrelationships between activities, and offered clarity about the risks and consequences in this process, as well as the preventive measures built collaboratively by the multidisciplinary team.

The prospects for the future are to expand this work with a broader perspective beyond those restricted to patient safety, encompassing administrative and clinical processes.

Our study has the following limitations. First, the study was finished during the height of the COVID-19 pandemic, when care teams were overwhelmed early in the pandemic. Second, changing the group protocol, which foresaw six participants per group for smaller groups due to emergencies was not anticipated. However, we did not expect as many staff to be absent at the same time. For future studies, increasing the number of participants to be interviewed is key. Finally, the FMEA method focused on a single component at a time and does not address the interrelated effects that can be affected by a shared cause resulting in multiple simultaneous failures. Also, the FMEA can be subjective depending on the team members” expertise and experience [29, 30].

## Conclusions

We found that the novel risk management program is highly effective in treating potential handoff process failures and sets the stage for introducing preventive decision-making interventions during patient handoffs between the OR and the ICU.

We observed that the training was very well received by the clinical teams and made sense to them. In addition, when comparing the qualitative themes that emerged before and after the training, we noticed a mature change in the terminology used, starting with the use of terms and concepts more appropriate to dynamic risk management and systems thinking. Participants recognized that the OR to ICU process involves many shifting patient safety risks, but also poses potential risks to the employees, the institution’s reputation, and can carry significant financial risks to the healthcare system.

We recommend that risk management analyses be used and applied following ISO 31000:2018 guidelines [8] to other clinical processes, and developed by professionals directly involved in the process under analysis, providing resources for the development of the method and the preventive measures identified by frontline clinical teams.

## Supplementary Material

mzae114_Supp

## Data Availability

Data cannot be shared for ethical/privacy reasons. The data underlying this article cannot be shared publicly due to privacy concerns regarding the individuals who participated in the study and the healthcare institution. The data will be shared upon reasonable request to the corresponding author.
