# Supplementary material for: Analyzing and mitigating the risks of patient harm during operating room to intensive care unit patient handoffs
Source: Int J Qual Health Care. 2024 Dec 19;37(1):mzae114. doi: 10.1093/intqhc/mzae114 (PMC11739622; doi:10.1093/intqhc/mzae114)
Supplement: mzae114_Supp [file mzae114_supp.zip › suppl_data/Suplementary Data.docx]

APPENDICES

## Project logo developed for communication with participants

A logo was developed for project communication so participants could quickly and easily identify that the messages related to the study.


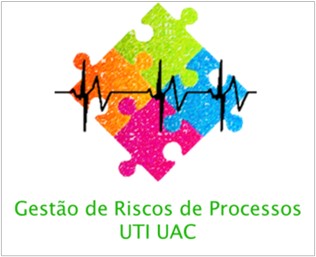


## Process representation exercise with health care providers using colored postcards


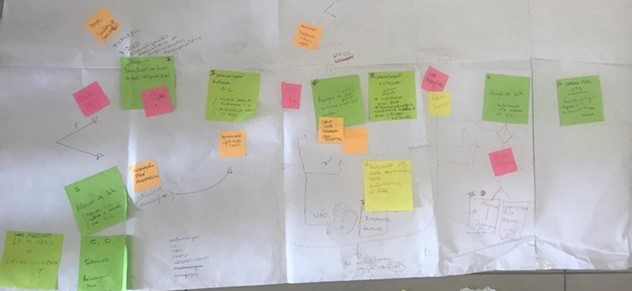


## Mapping the patient process during care transitions from the operating room (OR) to the intensive care unit (ICU)


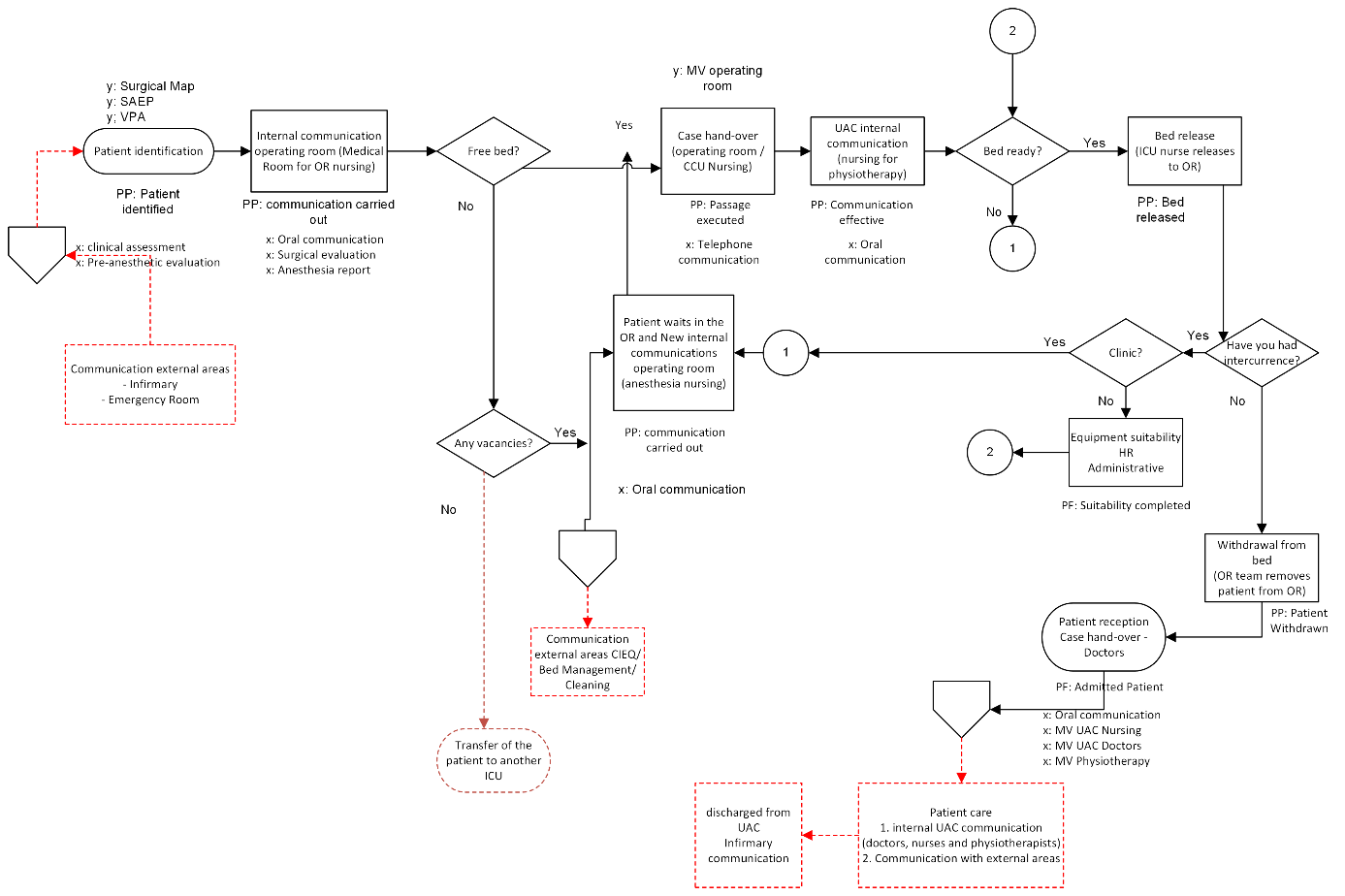


## The EQUATOR (Enhancing the QUAlity and Transparency Of health Research) Network

We used two EQUATOR Network guidelines (https://www.equator-network.org) for this research.

1. **COREQ**

| **Consolidated criteria for reporting qualitative studies (COREQ): 32-item checklist** | |
| --- | --- |
| **No Item** | **Guide questions/description** |
| **Domain 1: Research team and reflexivity** | |
| **Personal Characteristics** | |
| 1. Interviewer/facilitator | Which author/s conducted the interview or focus group? NRS |
| 2. Credentials | What were the researcher’s credentials? Statistics, MBA |
| 3. Occupation | What was their occupation at the time of the study?  Statistician |
| 4. Gender | Was the researcher male or female? Female |
| 5. Experience and training | What experience or training did the researcher have? 20 years in statistical research and process improvement |
| **Relationship with participants** | |
| 6. Relationship established | Was a relationship established prior to study commencement? No |
| 7. Participant knowledge of the interviewer | What did the participants know about the researcher? Before the survey, the participants did not know the researcher, who presented their credentials at the beginning of the survey. |
| 8. Interviewer characteristics | What characteristics were reported about the interviewer/facilitator? The interviewer had no professional or personal ties with the health professionals who took part in the research or with the hospital, thus remaining impartial. |
| **Domain 2: Study design** | |
| **Theoretical framework** | |
| 9. Methodological orientation and  Theory | What methodological orientation was stated to underpin the study? The study design used action-research methodology. |
| **Participant selection** | |
| 10. Sampling | How were participants selected? Convenience sample from among hospital employees. |
| 11. Method of approach | How were participants approached? face-to-face and by email. |
| 12. Sample size | How many participants were in the study? 100% of the health professional stakeholders who took part in the intervention (implementation of process risk management). Totaling 13 multidisciplinary healthcare providers |
| 13. Non-participation | How many people refused to participate or dropped out? Reasons? No participations refused to participate or dropped out. |
| **Setting** | |
| 14. Setting of data collection | Where was the data collected? In the hospital. |
| 15. Presence of non-participants | Was anyone else present besides the participants and researchers? No. |
| 16. Description of sample | What are the important characteristics of the sample? Health professional stakeholders who took part in the intervention (implementation of process risk management).  The study participants were composed of 85% women, 69% of the employees had university degrees (including 23% had PhD degrees), 72% worked exclusively at the ICHC-FMUSP hospital, and 77% were front line providers not in management positions.  The data were collected from February 1, 2020 and completed on April 30, 2020 |
| **Data collection** | |
| 17. Interview guide | Were questions, prompts, guides provided by the authors? Was it pilot tested? Yes. |
| 18. Repeat interviews | Were repeat interviews carried out? If yes, how many?  Yes, one interview was done before the intervention and one after the risk management intervention. |
| 19. Audio/visual recording | Did the research use audio or visual recording to collect the data? Yes, audio recording. |
| 20. Field notes | Were field notes made during and/or after the interview or focus group? Yes. |
| 21. Duration | What was the duration of the interviews or focus group?  60 minutes. |
| 22. Data saturation | Was data saturation discussed? No. |
| 23. Transcripts returned | Were transcripts returned to participants for comment and/or correction? No. |
| **Domain 3: analysis and findings** | |
| **Data analysis** | |
| 24. Number of data coders | How many data coders coded the data? Three. |
| 25. Description of the coding tree | Did authors provide a description of the coding tree? Yes. |
| 26. Derivation of themes | Were themes identified in advance or derived from the data? The themes were derived from the data. |
| 27. Software | What software, if applicable, was used to manage the data?  Iramuteq.^[[1]](#footnote-1)^ |
| 28. Participant checking | Did participants provide feedback on the findings? No. |
| 29. Quotations presented | Were participant quotations presented to illustrate the themes / findings? Was each quotation identified? Yes. |
| 30. Data and findings consistent | Was there consistency between the data presented and the findings? Yes. |
| 31. Clarity of major themes | Were major themes clearly presented in the findings? Yes. |
| 32. Clarity of minor themes | Is there a description of diverse cases or discussion of minor themes? Yes. |

1. Souza MAR, Wall ML, Thuler ACMC, Lowen IMV, Peres AM. The use of IRAMUTEQ software for data analysis in qualitative research. Rev Esc Enferm USP. 2018 Oct 4;52:e03353. Portuguese, English. doi: 10.1590/S1980-220X2017015003353. PMID: 30304198. [↑](#footnote-ref-1)
