# Supplementary figures and images for: Analyzing and mitigating the risks of patient harm during operating room to intensive care unit patient handoffs
Source: Int J Qual Health Care. 2024 Dec 19;37(1):mzae114. doi: 10.1093/intqhc/mzae114 (PMC11739622; doi:10.1093/intqhc/mzae114)

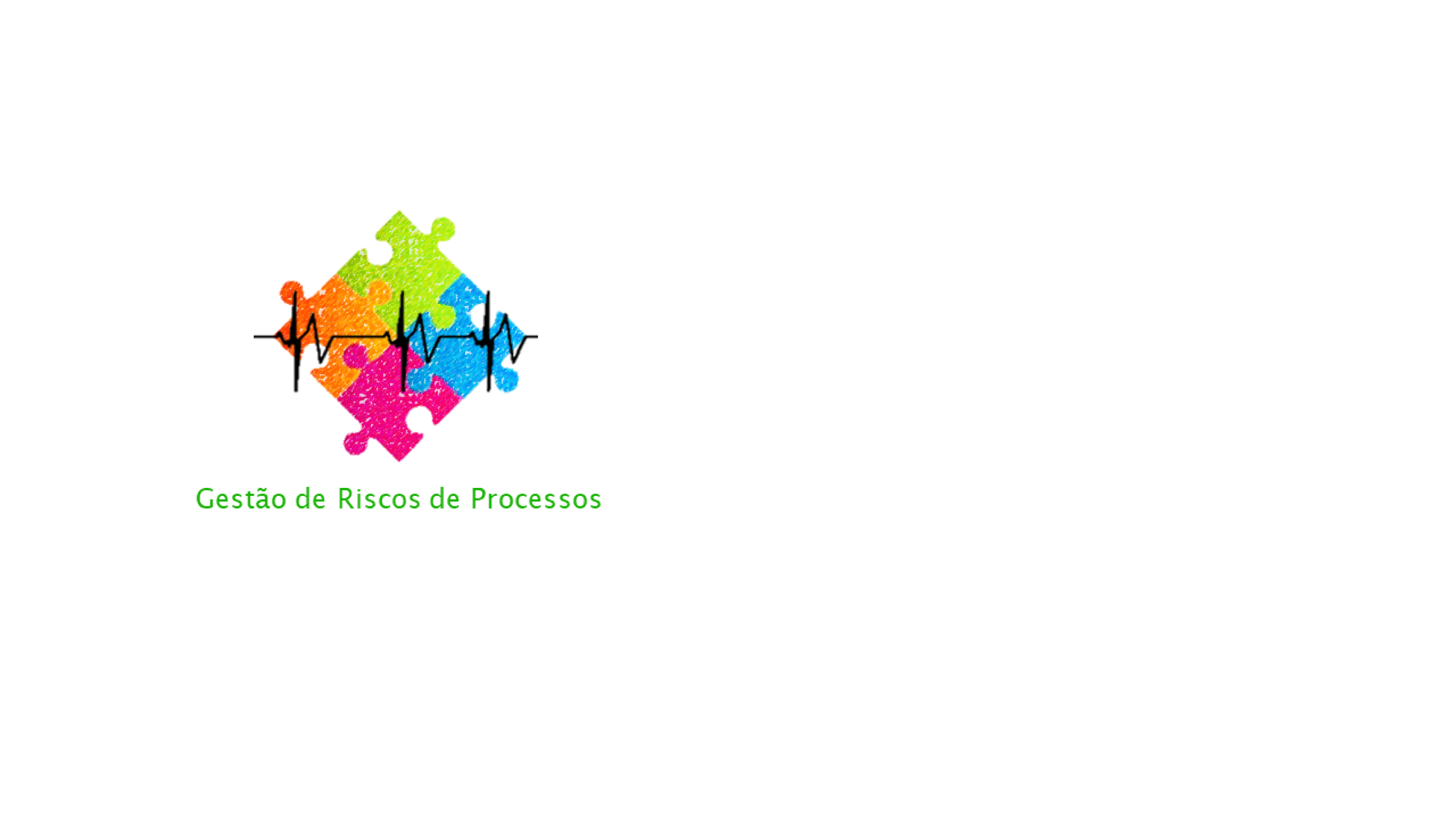

Supplement: mzae114_Supp [file mzae114_supp.zip › suppl_data/Supplementary Data 1 logo.tif]

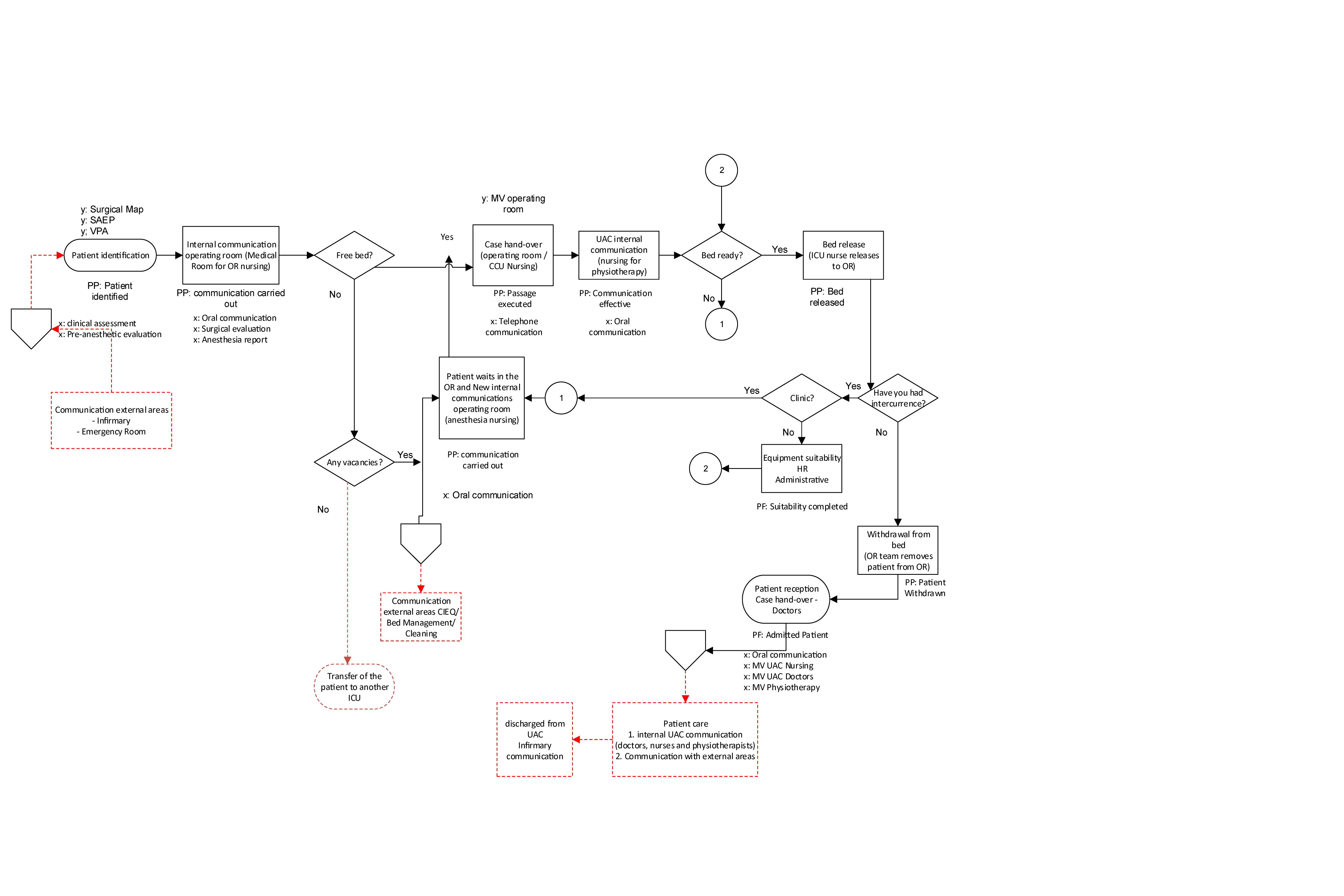

Supplement: mzae114_Supp [file mzae114_supp.zip › suppl_data/Supplementary Data 3 Mapping process.tif]
